# Fibroblast growth factor signaling and inhibition in non-small cell lung cancer and their role in squamous cell tumors

**DOI:** 10.1002/cam4.238

**Published:** 2014-04-08

**Authors:** Ravi Salgia

**Affiliations:** Section of Hematology/Oncology, Department of Medicine, University of ChicagoChicago, Illinois

**Keywords:** Angiogenesis inhibitors, fibroblast growth factors, non-small cell lung cancer, squamous cell carcinoma

## Abstract

With the introduction of targeted agents primarily applicable to non-small cell lung cancer (NSCLC) of adenocarcinoma histology, there is a heightened unmet need in the squamous cell carcinoma population. Targeting the angiogenic fibroblast growth factor (FGF)/FGF receptor (FGFR) signaling pathway is among the strategies being explored in squamous NSCLC; these efforts are supported by growth-promoting effects of FGF signaling in preclinical studies (including interactions with other pathways) and observations suggesting that FGF/FGFR-related aberrations may be more common in squamous versus adenocarcinoma and other histologies. A number of different anti-FGF/FGFR approaches have shown promise in preclinical studies. Clinical trials of two multitargeted tyrosine kinase inhibitors are restricting enrollment to patients with squamous NSCLC: a phase I/II trial of nintedanib added to first-line gemcitabine/cisplatin and a phase II trial of ponatinib for previously treated advanced disease, with the latter requiring not only squamous disease but also a confirmed *FGFR* kinase amplification or mutation. There are several ongoing clinical trials of multitargeted agents in general NSCLC populations, including but not limited to patients with squamous disease. Other FGF/FGFR-targeted agents are in earlier clinical development. While results are awaited from these clinical investigations in squamous NSCLC and other disease settings, additional research is needed to elucidate the role of FGF/FGFR signaling in the biology of NSCLC of different histologies.

## Introduction

Histologic determination in advanced non-small cell lung cancer (NSCLC) has only recently become a fundamental consideration in guiding treatment decisions 1. The most common histologic subtypes of NSCLC, which accounts for an estimated 85% of lung cancers, are adenocarcinoma (∼30–50% of cases), squamous cell carcinoma (∼30% of cases), and large cell carcinomas (∼10% of cases) 2. Historically, squamous cell carcinomas had been the predominant subtype but were supplanted by adenocarcinomas, likely reflecting changes related to the composition of cigarettes 2.

NSCLC-directed targeted therapies introduced into clinical practice over the past decade are mainly applicable to the treatment of patients with adenocarcinomas. These include the epidermal growth factor receptor (EGFR) tyrosine kinase inhibitors (TKIs) gefitinib (Iressa®, AstraZeneca; Wilmington, DE) 3 and erlotinib (Tarceva®, Genentech; South San Francisco, CA) 4 and the anaplastic lymphoma kinase (ALK) inhibitor crizotinib (Xalkori®, Pfizer; New London, CT) 5. Underlying aberrations conferring response to these agents (i.e., *EGFR* mutations and *ALK* gene rearrangements, the presence of which are to be confirmed by molecular analysis) are predominantly seen in adenocarcinomas 1,6. Additionally, the anti-vascular endothelial growth factor (VEGF) monoclonal antibody bevacizumab (Avastin®, Genentech; South San Francisco, CA) 7 is approved specifically for nonsquamous NSCLC because of heightened bleeding-related safety issues among patients with squamous tumors 8,9, an observation that has extended to some small molecule inhibitors, including sorafenib (Nexavar®, Bayer; Leverkusen, Germany) 10, sunitinib (SU11248, Sutent®, Pfizer; New London, CT) 11, and motesanib (Amgen; Thousand Oaks, CA) 12.

With the lack of applicability of the newest agents for treating NSCLC, squamous NSCLC poses unique challenges in the clinic and is being recognized as a subset with particularly high need for new therapies. Among tumors classified as squamous NSCLC, heterogeneity in angiogenic and proliferative behavior has been described 13. To date, identifying serum tumor markers and growth factors with prognostic relevance specifically in squamous NSCLC has proved to be an elusive goal 14. However, there is accumulating evidence that points toward a role for inhibiting the angiogenic fibroblast growth factor (FGF)/FGF receptor (FGFR) signaling pathway in squamous NSCLC 15–17. Following an overview of the FGF/FGFR signaling pathway, this article discusses key observations regarding its role in the development and progression of NSCLC and opportunities for its therapeutic inhibition in NSCLC, particularly for squamous cell disease.

## Overview of FGF and FGFRs

### Biology and hallmarks

**Figure 1:**
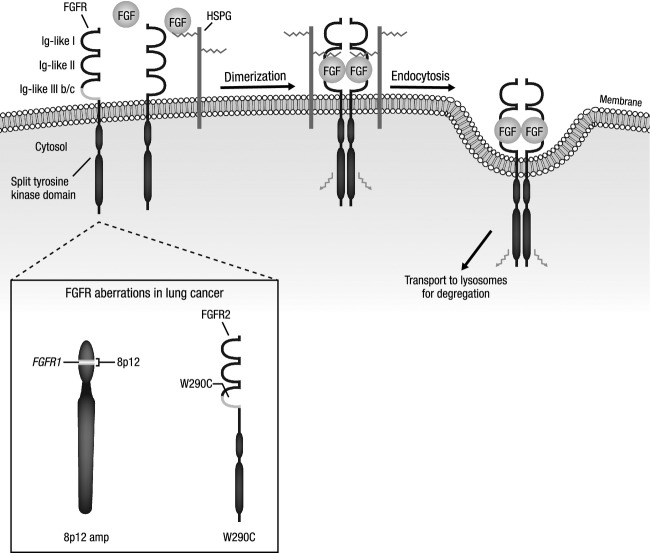
FGFR structure and function. FGFRs are single-pass transmembrane receptor tyrosine kinases consisting of an extracellular Ig-like domain and an intracellular split tyrosine domain. Upon ligand binding, FGFRs dimerize, resulting in transphosphorylation and activation of downstream signaling cascades. After activation, the receptor complex is internalized by endocytosis and degraded by lysosomes. Reproduced with permission from Wesche and colleagues 2011 18, *Biochem J*, 437:199-213 © the Biochemical Society. FGFR, fibroblast growth factor receptor; FGF, fibroblast growth factor; HSPG, heparan sulfate proteoglycan.

FGFs belong to a family of highly conserved polypeptide growth factors 18,19. Most of the FGFs have a similar internal core structure, consisting of six identical amino acid residues and 28 highly conserved residues, with 10 of the latter interacting with the FGFRs 19. Each of the four FGF tyrosine kinase receptors (FGFR1, FGFR2, FGFR3, and FGFR4) contains an extracellular component of three immunoglobulin-like domains (Ig-like I–III), a transmembrane domain, and an intracellular tyrosine kinase domain responsible for signal transmission to the cellular interior 18,19. Alternative splicing in Ig-like III of FGFR1 through three results in isoforms with varying degrees of binding specificity; FGFR IIIb and IIIc isoforms are mainly epithelial and mesenchymal, respectively 18,19. When FGFs bind to the FGFRs, dimerization results from a complex of two FGFs, two FGFRs, and two heparin sulfate chains (Fig. 1) and ultimately leads to FGFR activation, with the adaptor protein FGFR substrate two serving to recruit the Ras/mitogen-activated protein kinase (MAPK) and phosphoinositide-3 kinase (PI3K)/protein kinase B (Akt) pathways 18.

### Genetics of FGFRs

A total of 22 *FGF* genes have been identified in humans, of which the chromosomal locations have been established with one exception (*FGF16*) 19. Clustering within the genome (e.g., *FGF3*, *FGF4*, and *FGF19*, all on chromosome 11q13, and both *FGF6* and *FGF23* on chromosome 12p13) illustrates formation of the FGF family via gene and chromosomal duplication and translocation 19. *FGFR* mutations have been associated with developmental disorders and identified across a number of malignancies, including lung cancer (Table 1) 18. In addition to somatic *FGFR1* and *FGFR2* mutations (Table 1), *FGFR4* mutations have been observed in lung adenocarcinoma with a potential contributing role to carcinogenesis 20,21. In a Japanese study of *FGFR4* mutations and polymorphisms in surgically resected NSCLC, there were no *FGFR4* mutations in the analyzed samples per direct sequencing 22. However, when applying a genotyping assay, homozygous or heterozygous *FGFR4* Arg388 allele was present in 61.8% of patients.

**Table 1:** FGFR aberrations identified in human cancer.^1^

Cancer	Receptor	Aberration	Estimated prevalence	Association with other syndromes	Molecular consequence
Breast	FGFR1	8p11-12 amp	∼10% 18	Not known	Amplification of *FGFR1*
Bladder	FGFR3	R248C	5–20% 71–80	TDI	Enhanced kinase activity
	FGFR3	S249C	25–69% 71–82	TDI	Enhanced kinase activity
	FGFR3	G370/372C	2–9% 71–81	TDI	Enhanced kinase activity
	FGFR3	S371/373C	1–4% 71–73,76,79,80	TDI	Enhanced kinase activity
	FGFR3	Y373/375C	9–30% 71–81	TDI	Enhanced kinase activity
	FGFR3	G380/382R	<1–4% 71–73,80,82	ACH	Enhanced kinase activity
	FGFR3	A391/393E	<1–1% 71,73,75,81,82	CS	Enhanced kinase activity
	FGFR3	K650/652E/Q/M/T	<1–6% (E),	TDI, TDII, HCH, SADDAN, AN	Enhanced kinase activity
			<1–2% (Q),		
			1–3% (M),		
			<1% (T) 71,74–77,79,81,82		
Prostate	FGFR3	S249C	<1–6% 83,84	TDI	Enhanced kinase activity
	FGFR3	A391E	<1–2% 83,84	CS	Enhanced kinase activity
Endometrial	FGFR2	S252W	4–6% 85–87	AS	Altered ligand specificity
	FGFR2	P253R	3% 85	AS	Altered ligand specificity
	FGFR2	N549K	3–4% 86,87	Not known	Enhanced kinase activity
	FGFR2	K659N	1% 85–87	CR	Enhanced kinase activity
Lung	FGFR1	8p12 amp	11–20% (SSC) 35,88,89	Not known	Amplification of *FGFR1*
	FGFR2	W290C	2–3% 85,90,91	PS	Not known^2^
Rhabdomyosarcoma	FGFR4	N535K	3% 92	Not known	Enhanced kinase activity
	FGFR4	V550E	3% 92	Not known	Enhanced kinase activity
Multiple myeloma	FGFR3	t(4:14) trans	15–23% 93–96	Not known	Overexpression of FGFR3
	FGFR3	R248C	1–2% 93,97	TDI	Enhanced kinase activity
	FGFR3	K650/652M	<1–5% 93,98,99	TDI, SADDAN	Enhanced kinase activity
Brain	FGFR1	N56K	5% 100	Not known	Enhanced kinase activity
	FGFR1	K656E	NA	Not known	Enhanced kinase activity
Head and neck	FGFR3	R248C	5% 101	TDI	Enhanced kinase activity
	FGFR3	S249C	1% 102	TDI	Enhanced kinase activity
	FGFR3	G697C	NA	Not known	Enhanced kinase activity
Melanoma	FGFR2	I642V	1% 103	Not known	Reduced kinase activity
EMS	FGFR1	8p11-12 trans	100% 104	Not known	Constitutively active FGFR1-fusion proteins

FGFR, fibroblast growth factor receptor; amp, amplification; TDI/II, thanatophoric dysplasia I/II; ACH, achondroplasia; CS, Crouzon syndrome; HCH, hypochondroplasia; SADDAN, severe achondroplasia with developmental delay and acanthosis nigricans; AN, acanthosis nigricans; AS, Apert syndrome; CR, craniosynostosis; SCC, squamous cell carcinoma; PS, Pfeiffer syndrome; trans, translocation; NA, not available; EMS, 8p11 myeloproliferative disorder. The table, except for the column “Estimated prevalence” was reproduced with permission from Wesche and colleagues 2011 18, *Biochem J*, 437:199-213 © the Biochemical Society.

1 Includes only the aberrations identified in human tumor samples.

2 FGFR2 W290G forms ligand-independent dimers.

### Biologic effects of FGF signaling in normal physiology

FGF/FGFR signaling plays a role in stimulating cell proliferation and migration and promoting survival of various types of cells 18. Overall, FGFs are key contributors to not only angiogenesis but also organogenesis, including the formation of the heart, lungs, limbs, nervous system, and mammary and prostate glands 18.

## Role of the FGF Signaling Pathway in NSCLC

Serum basic FGF (bFGF) levels have been shown to be increased in the NSCLC population (including both squamous cell and adenocarcinoma histologies) relative to healthy controls 23,24. In the past decade, research to elucidate the role of the FGF signaling pathway in NSCLC proliferation and differentiation has intensified. In one preclinical study performed with this research question in mind, Kuhn and colleagues found that intracellular levels and mRNA expression of bFGF correlated with the proliferation rate of all three NSCLC cell lines evaluated and that intracellular bFGF appears to function as an intrinsic growth factor in the setting of NSCLC 25.

There is a substantial and growing body of literature to support that the FGF signaling pathway interacts with and influences other signaling pathways involved in the development and progression of NSCLC. For example, the VEGF and FGF/FGFR pathways have been shown to act synergistically in promoting tumor angiogenesis 26, while an upregulation of bFGF was recently proposed as one of the mechanisms by which the janus kinase 2/signal transducer and activator of transcription 3 (JAK2/STAT3) pathway mediates tumor angiogenesis in NSCLC 27. One in vitro series involving a newly developed squamous NSCLC line (SCC-35), in which there was a highly significant correlation between the overexpression of FGF3 and EGFR, supports that co-overexpression of both growth factors may be implicated in the pathogenesis of lung carcinoma 28. Furthermore, cancer-associated fibroblasts and the FGF/FGFR signaling pathway have been implicated in the development of intrinsic and acquired resistance to EGFR TKIs in patients with NSCLC 29–32.

Interestingly, there appear to be some FGF/FGFR signaling pathway-related distinctions between NSCLC cases of squamous cell versus adenocarcinoma histology 15–17,33,34. Recently, researchers from the Dana–Farber Cancer Institute (DFCI) and the Broad Institute described a high prevalence of *FGFR1* amplification specifically in squamous NSCLC, with amplification of a region of chromosome segment 8p11-12 (which includes the *FGFR1* gene) in 21% of squamous tumors versus 3% of adenocarcinomas (*P* < 0.001) 15. Similarly, a previously published German study had identified frequent and focal *FGFR1* amplification in squamous NSCLC but not other histologic subtypes of lung cancer 16, while Japanese researchers have since reported a significantly higher rate of increased *FGFR1* copy number in surgically resected squamous versus nonsquamous NSCLC (41.5% vs. 14.3%; *P* = 0.0066) 17. However, there have been some reports to the contrary; for example, a recent German study designed to further elucidate the relevance of FGFR1 in lung cancer found that the proportion of samples displaying ≥4 copies of the *FGFR1* gene was numerically but not statistically higher for squamous versus adenocarcinoma histology (10.5% vs. 4.7%; *P* = 0.278) 35.

Accumulating evidence points to a role for FGF signaling in the disease invasion and metastasis characteristic of NSCLC 36,37. In a recent study focused on identifying angiogenesis-related microRNAs (miRs) altered in NSCLC, one miR (miR-155) was found to be significantly correlated with FGF2 in the overall cohort (*r* = 0.17; *P* = 0.002), but even more strongly in the subset with nodal disease (*r* = 0.34; *P* < 0.001) 36.

FGFs/FGFRs have been identified as potential predictive and prognostic markers in NSCLC. In a number of studies, pretreatment bFGF levels have been correlated with prognosis in the NSCLC population 38–43. In addition, recent evidence supports *FGFR1* amplification as an independent negative prognostic factor (while exhibiting a dose-dependent association with cigarette smoking) in patients with squamous NSCLC 44. A series of studies by Brattström and colleagues yielded mixed findings, with elevated serum bFGF levels reported as a favorable prognostic factor in an early series 45, but as a negative prognostic factor in subsequent reports 38,39. One of the studies was based on samples from 58 patients with surgically resected NSCLC, in whom a number of variables (including bFGF, as well as tumor volume, platelet counts, and serum VEGF levels) were significant prognostic factors on univariate analysis, whereas significance was retained only for bFGF on multivariate analysis 38. There was a significant correlation between bFGF and disease recurrence (*r* = 0.34; *P* = 0.01), with rates of 78% and 40% for patients with elevated and normal bFGF levels, respectively. Additionally, this study found a significant correlation between bFGF levels and VEGF levels (*r* = 0.44; *P* < 0.001) and that the combination of growth factors was a significant prognostic factor on univariate but not multivariate analysis, although conclusions were confounded by the presence of elevated levels of both bFGF and VEGF in only six patients. In a Japanese retrospective analysis of predictors of long-term survival among 71 patients with surgically resected NSCLC of adenocarcinoma or squamous histology, mean bFGF levels were significantly higher in cases of metastatic nodal involvement and high levels were most strongly correlated with poor prognosis in patients also exhibiting high VEGF levels (*P* < 0.0001) 42. Per multivariate analysis, bFGF and VEGF levels were each independent prognostic factors regardless of histology. Adding complexity to the topic of FGF as a prognostic factor in NSCLC, the implications of increased FGF expression have been shown to differ based on its presence in tumor cells (negative prognostic marker) versus stroma (favorable prognostic marker) 46,47, with stromal expression postulated to inhibit NSCLC progression 48. From a predictive biomarker standpoint, data on the contribution of baseline FGF levels on response to treatment for NSCLC have been mixed, with some but not all studies supporting a potential role for FGF to predict for treatment outcomes in various settings (including but not limited to antiangiogenic regimens) 49–52.

## Therapeutic Inhibition of FGF/FGFR Signaling

### Preclinical observations in NSCLC

A number of preclinical observations collectively suggest that FGF/FGFR signaling may be exploited as a therapeutic target in the NSCLC population. In the aforementioned DFCI study, in which 21% of squamous tumors exhibited *FGFR1* amplification, cell growth inhibition of an NSCLC line with focal *FGFR1* amplification was demonstrated via FGFR1-specific small hairpin ribonucleic acids (shRNAs) or small molecule inhibitors 15. Earlier preclinical series had supported inhibitory activity against NSCLC for a number of different anti-FGF/FGFR therapies, including a bFGF-neutralizing monoclonal antibody, antisense oligonucleotides, or bFGF antisense cDNA-expressing vector in one study 25 and a dominant-negative FGFR1 IIIc-green fluorescent protein fusion protein or small molecule inhibitors in another study 53. Additional preclinical data have described the antiangiogenic and antitumor activities of individual multitargeted small molecule inhibitors—specifically those for which the targets include FGF/FGFRs—against NSCLC; these include cediranib (Recentin™, AstraZeneca; Wilmington, DE) 54, nintedanib (BIBF 1120, Boehringer Ingelheim; Ingelheim, Germany) 55, pazopanib (Votrient™, GlaxoSmithKline; London, UK) 56, ponatinib (Iclusig®, ARIAD Pharmaceuticals, Inc, Cambridge, MA) 57, and a number of other investigational agents 16,58–61. Of note, inhibiting bFGF has been shown to increase the secretion of VEGF in NSCLC lines, supporting a therapeutic role for bFGF inhibition as a component of a multitargeted approach that also includes VEGF inhibition 62.

### Clinical trials of FGF-targeting agents in NSCLC

Ongoing clinical trials of FGFR-inhibiting multitargeted tyrosine kinases in advanced squamous NSCLC or advanced NSCLC in general, including but not limited to squamous histology, are summarized in Table 2. Two multitargeted agents are being evaluated in a squamous-exclusive NSCLC population: (1) nintedanib, an inhibitor of VEGFR1 through 3, FGFR1 through 4, platelet-derived growth factor receptor (PDGFR) *α* and *β*, fms-related tyrosine kinase 3 (FLT-3), and members of the src family 55 and (2) ponatinib, a breakpoint cluster (BCR)–c-abl oncogene 1, nonreceptor tyrosine kinase (ABL) inhibitor (approved in December 2012 for treating two types of leukemia) that has also been shown to inhibit the four FGFRs, fueling research to determine its therapeutic potential as an FGFR inhibitor 57. In an ongoing placebo-controlled phase I/II study, nintedanib is being added to gemcitabine/cisplatin as first-line treatment of advanced or recurrent NSCLC specifically of squamous histology (NCT01346540). An estimated 165 patients will be enrolled, with primary outcomes of adverse events and dose-limiting toxicities in phase I and progression-free survival in phase II. In a completed, open-label, phase I trial (*N* = 26, including three with squamous histology) of first-line nintedanib in combination with carboplatin/paclitaxel in advanced NSCLC, among seven patients with a confirmed partial response, two had squamous histology and one had mixed large cell/squamous histology 63. The most commonly reported adverse events (occurring in ≥10% of patients) related to nintedanib were diarrhea (53.8%), fatigue (50.0%), and nausea (46.2%). For ponatinib, a phase II trial is underway in patients with advanced squamous NSCLC that had progressed after the most recent treatment regimen, also requiring that patients have confirmed *FGFR* kinase amplification or mutation per genotyping (NCT01761747). This trial has a target accrual of 40 patients and a primary endpoint of response. Orantinib (formerly TSU-68 and SU6688; Taiho Pharmaceutical Co. Ltd, Tokyo, Japan), an oral TKI that targets VEGFR2, PDGFR*β*, and FGFR1, was evaluated in a phase I trial (*N* = 37, including five patients with squamous NSCLC) in combination with carboplatin/paclitaxel as first-line therapy for advanced NSCLC, with 13 partial responses observed among 33 evaluable patients 64. It was not specified as to whether any of these responses were in the squamous participants, and there are no known active clinical trials of this agent in advanced NSCLC as of this writing.

**Table 2:** Ongoing trials^1^ of multitargeted antiangiogenic tyrosine kinase inhibitors in squamous NSCLC

Agent	Phase	Regimen	Trial identifier
General NSCLC (including squamous)^2^			
Cediranib	III	Cediranib + first-line paclitaxel/carboplatin for advanced or metastatic NSCLC	NCT00795340
Nintedanib (BIBF 1120)	III	Nintedanib + second-line docetaxel for locally advanced and/or metastatic, or recurrent NSCLC	NCT00805194
Pazopanib	II/III	Pazopanib as maintenance therapy after first-line chemotherapy for advanced NSCLC	NCT01208064
	II	Pazopanib as second-line therapy after progression on bevacizumab-containing first-line therapy	NCT01262820
	II	Pazopanib + erlotinib as second- or third-line therapy for advanced NSCLC	NCT01027598
	II	Pazopanib + paclitaxel as first-line therapy for advanced NSCLC	NCT01179269
	I	Pazopanib + vinorelbine in metastatic NSCLC or breast cancer	NCT01060514
Dovitinib	II	Dovitinib after recent anti-VEGF therapy for advanced NSCLC or advanced colorectal cancer	NCT01676714
Squamous-exclusive NSCLC			
Ponatinib	II/III	Ponatinib for progressive squamous NSCLC or head and neck cancers with FGFR kinase alterations	NCT01761747
Nintedanib (BIBF 1120)	I/II	Nintedanib + first-line gemcitabine/cisplatin for advanced or recurrent squamous NSCLC	NCT01346540

NSCLC, non-small cell lung cancer; VEGF, vascular endothelial growth factor; FGFR, fibroblast growth factor receptor.

1 Includes trials indexed on ClinicalTrials.gov with a status of recruiting, not yet recruiting, or active, not recruiting, as of September 2013.

2 Phase I and II trials are included only for agents that have not reached phase III development for advanced NSCLC.

As shown in Table 2, two phase III trials have been initiated in NSCLC populations without exclusion of squamous cell histology, one of nintedanib plus docetaxel as second-line therapy in advanced or recurrent NSCLC (LUME-Lung 1 [NCT00805194]) and the other of cediranib in combination with first-line paclitaxel/carboplatin for advanced NSCLC (CAN-NCIC-BR29 [NCT00795340]). Results of LUME-Lung 1 show improvement in the primary outcome of progression-free survival with nintedanib/docetaxel versus placebo/docetaxel in the entire study population (median, 3.4 vs. 2.7 months; *P* = 0.0019) as well as in the histologic subsets with squamous disease (*P* = 0.02) or adenocarcinoma (*P* = 0.02) 65. Significant improvement in overall survival (OS) was also observed in the nintedanib group among patients with adenocarcinoma histology (median, 12.6 vs. 10.3 months with placebo plus docetaxel; *P* = 0.0359). Cediranib primarily targets VEGFR2 but has demonstrated some inhibitory activity against FGF-induced proliferation, albeit 275-fold less selective than its inhibition of VEGF-induced proliferation 54. A prior phase II trial (CAN-NCIC-BR24) found that cediranib (using a higher dose than in the phase III CAN-NCIC-BR29 trial above) plus paclitaxel/carboplatin was not tolerable. However, compared with other histologies, the squamous participants did not have an increased risk of severe pulmonary hemorrhage or adverse efficacy outcomes, which included the primary endpoint of progression-free survival 66. Regarding new-onset cavitation, 10 of 40 cases among cediranib recipients and seven of 23 cases among placebo recipients were in patients with squamous tumors.

The EGFR-directed monoclonal antibody cetuximab (ERBITUX®, ImClone; New York, NY) 67 is another targeted therapy that is currently under clinical evaluation for squamous NSCLC. A phase II trial investigated first-line cetuximab in combination with platinum-based chemotherapy in advanced or recurrent NSCLC (eLung [NCT00828841]; squamous or nonsquamous disease), with OS as the primary endpoint. Presented results showed that median OS with cetuximab-containing chemotherapy was significantly longer in patients with nonsquamous versus squamous disease (9.9 vs. 8.7 months; *P* = 0.0082) 68. A phase III study is currently recruiting patients with advanced NSCLC of any histology (including squamous) to receive carboplatin/paclitaxel with or without bevacizumab and/or cetuximab (NCT00946712).

Finally, there are ongoing clinical investigations of other FGF/FGFR-targeted agents in advanced malignancies, although not specific to NSCLC or squamous NSCLC. The pan-FGFR inhibitors AZD4547 (AstraZeneca; Wilmington, DE; NCT01213160) and BGJ398 (Novartis; Cambridge, MA; NCT01004224; NCT01697605) are being evaluated in a phase I trial for advanced solid tumors; for BGJ398, eligibility criteria include confirmed FGFR-related alterations. A nonrandomized phase II trial of AZD4547 monotherapy is enrolling previously treated patients with *FGFR1*-amplified advanced squamous NSCLC (or *FGFR1*-amplified advanced breast cancer or *FGFR2*-amplified advanced esophagogastric cancer), with serial biopsies being performed to assess molecular effects (NCT01795768) 69. Results are awaited from a phase I trial of the FGF ligand trap FP-1039 (FivePrime Therapeutics; South San Francisco, CA) in unresectable locally advanced or metastatic solid tumors (NCT00687505).

Future directions include studies to assess anti-FGF/FGFR agents in resectable disease (e.g., in combination with chemotherapy and/or radiation in the adjuvant setting), or even as a chemoprevention strategy 70. Clinical trials to date have only investigated the efficacy of anti-FGF/FGFR agents in advanced NSCLC. Given the potential role of the FGF/FGFR signaling pathway in the pathogenesis of NSCLC, inhibition of this pathway in the adjuvant setting could provide benefit, especially for patients with squamous disease.

## Conclusions

While there have been several molecularly targeted agents developed for the treatment of nonsquamous NSCLC, there appears to be a unique opportunity to develop anti-FGF/FGFR-based regimens for the treatment of NSCLC of squamous histology. Recent research findings supporting a propensity for squamous NSCLC to exhibit increased *FGFR1* gene amplification strengthen the rationale for this novel approach. Multitargeted small molecule inhibitors that inhibit FGFR along with other angiogenic pathways/receptors are the most advanced in clinical development, although none have yet to reach phase III evaluation in squamous-exclusive NSCLC study populations. Further research efforts are needed to more fully characterize the manner by and degree to which FGF signaling influences the underlying biology of specific NSCLC histologies.
